# Prognostic Significance of Baseline Lean Tissue Mass Percentage in Incident Peritoneal Dialysis Patients

**DOI:** 10.1016/j.xkme.2026.101260

**Published:** 2026-01-10

**Authors:** Lixing Xu, Jack Kit-Chung Ng, Gordon Chun-Kau Chan, Winston Wing-Shing Fung, Kai-Ming Chow, Cheuk-Chun Szeto

**Affiliations:** 1 Department of Medicine & Therapeutics, Carol & Richard Yu Peritoneal Dialysis Research Centre, Prince of Wales Hospital; 2 Faculty of Medicine, Li Ka Shing Institute of Health Sciences, The Chinese University of Hong Kong, Shatin, New Territories, Hong Kong, China

**Keywords:** Kidney failure, malnutrition, sarcopenia, frailty

## Abstract

**Rationale & Objective:**

Bioimpedance spectroscopy is a convenient method to measure lean tissue mass (LTM), which is commonly taken as the skeletal muscle mass, in peritoneal dialysis (PD) patients. We investigated the prognostic significance of LTM as the percentage of body weight (%LTM) and as the marker of sarcopenia in incident PD patients.

**Study Design:**

A retrospective review of a prospective cohort.

**Setting & Participants:**

349 incident PD patients in a single center.

**Predictor:**

Baseline %LTM.

**Outcomes:**

Patient survival, technique survival, peritonitis-free survival, peritonitis rate, hospitalization rate, and the duration of hospitalization.

**Analytical Approach:**

Time-to-event survival analyses; linear regression for hospitalization.

**Results:**

The 5-year patient survival rates were 50.2%, 55.3%, 61.0%, and 72.6% for patients with %LTM quartiles I-IV, respectively (log-rank test; *P* = 0.02). Multivariable Cox regression analysis confirmed that baseline %LTM was associated with patient survival (adjusted HR, 0.982; 95% CI, 0.966-0.999; *P* = 0.04). Baseline %LTM was also associated with peritonitis-free survival (adjusted HR, 0.983; 95% CI, 0.968-0.998; *P* = 0.031), but not technique survival. %LTM was also significantly and inversely associated with the hospitalization rate (*P* = 0.002) and the duration of hospitalization (*P* = 0.02). Yet, %LTM was unrelated to the 5-year technique survival (*P* = 0.29) and the peritonitis rate (*P* = 0.15). The number of hospital admissions was 2.72, 2.39, 2.36, and 1.67 per year of follow-up for quartiles I-IV of baseline %LTM (Jonckheere-Terpstra test; *P* = 0.002), and the duration of hospital stay was 23.31, 20.84, 22.27, and 13.96 d/y, respectively (*P* = 0.02).

**Limitations:**

Observational study with baseline measures only.

**Conclusions:**

Baseline %LTM as measured using bioimpedance spectroscopy is associated with patient survival, peritonitis-free survival, the number of hospital admissions, and the duration of hospital stay in incident PD patients.

## Plain-Language Summary

Muscle wasting is a common problem in dialysis patients. In patients starting peritoneal dialysis, we measured their lean tissue mass using bioimpedance spectroscopy. We found that patients with higher percentages of lean tissue mass had better survival rates over 5 years, fewer and shorter hospital stays, and a lower risk of infection complications (peritonitis). Overall, measuring muscle mass in new peritoneal dialysis patients can help predict who is likely to do better with treatment and have fewer hospital problems, suggesting that monitoring and possibly improving muscle mass could be important for patient care.

Peritoneal dialysis (PD) is a life-saving treatment option for patients with kidney failure who require dialysis. PD offers several distinct advantages as compared with hemodialysis, such as its use a home-based therapy, lower cost, and better preservation of residual kidney function.^1^ Although peritonitis has long been the Achilles’ heel of PD, cardiovascular disease and malnutrition are now recognized as important contributing factors to the outcome of PD patients.^2^

Sarcopenia is characterized by low muscle mass and muscle strength in patients and can be caused by several factors, such as chronic inflammation and protein-energy wasting.^3^ It is a common and serious problem in PD patients and is associated with higher rates of all-cause mortality.^4^^,^^5^ Accurate assessment of muscle mass is therefore an important component in the clinical care of PD patients. Many methods are available for the evaluation of muscle mass, but bioimpedance spectrometry (BIS), also called bioelectrical impedance analysis, has emerged as the widely adopted noninvasive method. BIS offers a quick and cost-effective way to estimate the body composition of PD patients. The lean tissue mass (LTM) determined using BIS is often considered to represent skeletal muscle mass.^6^ However, there is no universally accepted cutoff value of LTM for the diagnosis of sarcopenia.

Although BIS has many advantages, it is subjected to several limitations. One notable concern is that the accuracy of LTM measurements may be affected by hydration status,^7^ whereas overhydration (OH) is a common issue in PD patients.^8^ In short, the presence of fluid overload may lead to an overestimation of the muscle mass when using BIS. To address this problem, we propose to use the percentage of LTM (%LTM). Because the OH volume measured using BIS is also positively correlated with body weight,^9^ expressing LTM as a percentage of body weight may remove the interference of OH and potentially offer an accurate way to evaluate the muscle build of PD patients.

Despite theoretical advantages, few studies have investigated the prognostic values of %LTM in PD patients. In the present study, we investigated the roles of baseline %LTM as the prognostic indicator of Chinese PD patients.

## Patients and Methods

The study was approved by the Joint Chinese University Hong Kong-New Territories East Cluster Clinical Research Ethics Committee (approval no.: CRE-2023.363). All procedures in this study followed the guidelines outlined in the Declaration of Helsinki.

### Patient Selection

This study is a retrospective analysis of a prospective observational cohort of incident adult PD patients from September 2009 to September 2023 at a single center. Patients who were scheduled for living donor kidney transplants or transferal to other renal centers in 6 months, and those with a pacemaker or metallic prosthesis, were excluded from this study. After written informed consent, baseline multifrequency BIS, dialysis adequacy, and other laboratory tests were performed around 4 weeks after patients became stable receiving PD.

### Multifrequency BIS

As described previously,^10^ we employed a BIS study to assess the LTM of PD patients. In brief, electrodes were attached to the patient’s right hand and right foot while in a supine position. The Body Composition Monitor (Fresenius Medical Care) was used to measure LTM. %LTM was computed using LTM and body weight. We also assessed adipose tissue mass, volume of OH, total body water, extracellular water, and intracellular water. The extracellular-to-intracellular fluid volume ratio was computed accordingly. All body composition measurements were conducted during PD fluid dwell because our previous study showed that peritoneal dialysate had minimal effect on measurements.^11^

### Dialysis Adequacy and Other Laboratory Indices

Assessment of dialysis adequacy was performed by 24-hour dialysate and urine collection as previously described,^12^ with the total weekly Kt/V calculated using the standard formula. The residual glomerular filtration rate was determined by the mean of the 24-hour urinary urea and creatinine clearances.^13^ Serum albumin levels were measured using the bromocresol purple method.^14^ Other laboratory tests, including routine biochemistry, hemoglobin levels, serum iron, total iron-binding capacity, and ferritin levels, were performed as part of the routine clinical care.

### Follow-up and Outcome Parameters

This study cohort was observed until December 31, 2023. During the follow-up period, clinical management was determined by the responsible individual clinician who was not influenced by the study. The primary outcome measures of this study include patient survival and technique survival. Secondary outcome measures include peritonitis-free survival, peritonitis rate, number of hospital admissions, and the total duration of hospitalization. The peritonitis rate was calculated as the number of peritonitis episodes per patient-year as previously described.^15^ In this study, technique failure was defined as transferal to hemodialysis, receiving a kidney transplant, or death, whereas transferal to other dialysis centers and recovery of kidney function were considered as censoring events.

### Statistical Analysis

Statistical analysis was performed using the software SPSS (version 28.0; IBM Corporation) and GraphPad Prism (version 10.1.1; GraphPad Software). Data are presented as mean ± standard deviation unless otherwise stated. Correlation between parameters was explored by the Pearson or Spearman correlation coefficient as appropriate. The Kaplan-Meier method was used to describe the patient, technique, and peritonitis-free survival rates. Patients were grouped in quartiles according to their baseline %LTM values, and the survival rates were compared using the log-rank test. Univariate Cox regression for patient, technique, and peritonitis-free survival was performed for all baseline clinical and biochemical parameters. Multivariable Cox regression models were then constructed from variables with *P* < 0.1 in the univariate analysis to identify predictors of patient and peritonitis-free survival. In the Cox regression models, we included %LTM and other variables that had *P* values < 0.1 in the univariate analysis. The backward elimination method was used to determine the risk factors as many baseline parameters had intrinsic correlation with baseline %LTM. The peritonitis rate, number of hospital admissions, and duration of hospitalization between %LTM quartiles were compared using the Jonckheere-Terpstra test. A *P* value of <0.05 was taken as statistically significant. All probabilities were 2 tailed.

## Results

We studied 349 incident PD patients. Their baseline clinical and demographic information are summarized in Table 1. The results of multifrequency BIS as well as other baseline biochemical characteristics are summarized in Table 2. The average LTM was 45.05 ± 9.22 kg for male and 32.17 ± 6.85 kg for female patients (*P* < 0.001). The average %LTM was 66.72% ± 12.48% and 58.87% ± 13.54% for male and female patients, respectively (*P* < 0.001).

**Table 1:** Baseline Demographic and Clinical Characteristics

Characteristics	No. of Patients (N = 349)
Sex (male:female)	200:149
Age (y)	58.5 ± 11.7
Height (cm)	161.9 ± 8.5
Body weight (kg)	63.0 ± 13.8
Body mass index (kg/m^2^)	24.0 ± 4.2
Blood pressure (mm Hg)	
Systolic	135.7 ± 23.6
Diastolic	73.2 ± 14.9
Renal diagnosis	
Diabetic nephropathy	167 (47.9%)
Glomerulonephritis	78 (22.3%)
Hypertension	33 (9.5%)
Urological problem	13 (3.7%)
Polycystic kidney disease	15 (4.3%)
Others or unknown	43 (12.3%)
Major comorbid conditions	
Diabetes mellitus	199 (57.0%)
Coronary artery disease	92 (26.4%)
Cerebrovascular disease	80 (22.9%)
Peripheral vascular disease	24 (6.9%)
Charlson comorbidity score	6.1 ± 2.6
Type of PD	
Machine-assisted automated PD	72 (19.7%)
Low GDP solution	36 (10.3%)

*Note*: Continuous variables are presented as mean ± standard deviation.

Abbreviations: GDP, glucose degradation products; PD, peritoneal dialysis.

**Table 2:** Baseline Bioimpedance and Biochemical Information

Characteristics	No. of Patients (N = 349)
Bioimpedance spectroscopy	
LTM (kg)	39.6 ± 10.5
%LTM	63.4 ± 13.5
ATM (kg)	19.4 ± 10.6
Overhydration (L)	3.8 ± 3.0
E/I ratio	1.0 ± 0.2
Hemoglobin (g/dL)	9.5 ± 1.4
Serum albumin (g/L)	34.6 ± 4.9
Fasting plasma glucose (mmol/L)	6.0 ± 1.9
Lipid profile (mmol/L)	
Total cholesterol	4.8 ± 1.4
LDL cholesterol	2.7 ± 1.2
HDL cholesterol	1.3 ± 0.4
Triglyceride	1.7 ± 1.1
Total weekly Kt/V	2.2 ± 0.6
Residual GFR (mL/min/1.73 m^2^)	3.9 ± 2.8
Iron profile	
Plasma iron (μmol/L)	13.3 ± 5.4
Plasma TIBC (μmol/L)	38.9 ± 7.7
Iron saturation (%)	0.4 ± 0.2
Serum ferritin (ng/mL)	1,194.5 ± 1,027.4

*Note*: Continuous variables are presented as mean ± standard deviation.

Abbreviations: ATM, adipose tissue mass; E/I ratio, extracellular-to-intracellular fluid volume ratio; GFR, glomerular filtration rate; HDL, high-density lipoprotein; LDL, low-density lipoprotein; LTM, lean tissue mass; %LTM, lean tissue mass percentage; TIBC, total iron-binding capacity.

### Correlation With Baseline Characteristics

The relations between baseline %LTM and other baseline clinical and biochemical parameters are summarized in Table 3. In essence, adipose tissue mass has a significant inverse correlation with %LTM (r = −0.876; *P* < 0.001) and a less substantial correlation with LTM (r = −0.294; *P* < 0.001). There were significant but modest inverse correlations between %LTM and age, body weight, body mass index, Charlson comorbidity score, and the extracellular-to-intracellular fluid volume ratio, but not the OH volume.

**Table 3:** Correlation Between Baseline Bioimpedance Spectroscopy Measurements and Other Clinical and Biochemical Parameters

Characteristics	LTM		%LTM		ATM	
	r	*P* Values	r	*P* Values	r	*P* Values
Age (y)	−0.205	<0.001	−0.244	<0.001	0.128	0.03
Height (cm)	0.644	<0.001	0.230	<0.001	0.012	0.83
Weight (kg)	0.574	<0.001	−0.251	<0.001	0.556	<0.001
BMI (kg/m^2^)	0.320	<0.001	−0.446	<0.001	0.675	<0.001
Blood pressure (mm Hg)						
Systolic	0.017	0.79	−0.130	0.03	0.128	0.03
Diastolic	0.105	0.11	0.083	0.18	−0.035	0.62
Charlson comorbidity score^a^	−0.058	0.45	−0.219	<0.001	0.178	0.003
Other bioimpedance spectroscopy measurements						
Overhydration volume (L)	0.286	<0.001	−0.087	0.17	−0.015	0.83
E/I ratio	−0.265	<0.001	−0.586	<0.001	0.347	<0.001
Hemoglobin (g/dL)	−0.031	0.65	0.032	0.70	−0.013	0.83
Serum albumin (g/L)	0.025	0.71	0.000	0.99	0.157	0.01
Fasting plasma glucose (mmol/L)^a^	−0.094	0.24	−0.143	0.45	0.096	0.19
Lipid profile (mmol/L)						
Total cholesterol	−0.055	0.56	0.068	0.29	−0.118	0.12
LDL^a^	−0.016	0.80	0.048	0.62	−0.053	0.53
HDL^a^	−0.098	0.24	0.189	0.01	−0.279	<0.001
Triglyceride^a^	−0.119	0.13	−0.126	0.08	0.112	0.13
Total weekly Kt/V^a^	−0.168	0.04	0.002	0.99	−0.023	0.83
Residual GFR (mL/min/1.73 m^2^)^a^	0.320	<0.001	0.024	0.84	0.130	0.12
Iron profile						
Plasma iron (μmol/L)	0.046	0.60	0.187	0.01	−0.192	0.01
Plasma TIBC (μmol/L)	0.047	0.60	−0.002	0.99	0.106	0.15
Iron saturation (%)^a^	0.057	0.56	0.185	0.01	−0.219	0.002
Serum ferritin (pmol/L)^a^	−0.041	0.64	0.029	0.79	−0.091	0.23

*Note*: *P* values were adjusted using the Benjamini-Hochberg method; r represents correlation coefficient. Those parameters other than that denoted using the footnote were assessed using Pearson coefficient.

Abbreviations: ATM, adipose tissue mass; BMI, body mass index; E/I ratio, extracellular-to-intracellular fluid volume ratio; GFR, glomerular filtration rate; HDL, high-density lipoprotein; LDL, low-density lipoprotein; LTM, lean tissue mass; %LTM, lean tissue mass percentage; TIBC, total iron-binding capacity.

a Parameters assessed using Spearman coefficient.

### Patient Survival

**Figure 1:**
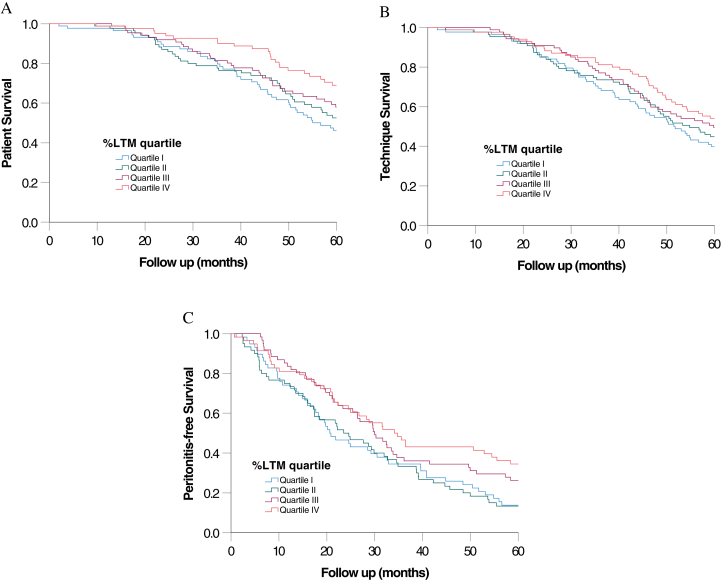
Kaplan-Meier plots of (A) patient survival, (B) technique survival, and (C) peritonitis-free survival. Patients were divided into quartiles according to the baseline lean tissue mass percentage (%LTM), with quartile I having the lowest values. Data were compared using the log-rank test.

The patients were followed for an average of 82.1 ± 54.0 months. During this period, 288 patients died. Their causes of death were nonperitonitis infection (114 cases), myocardial infarction (67 cases), peritonitis (29 cases), stroke (27 cases), sudden cardiac death (22 cases), termination of dialysis (11 cases), malignancy (9 cases), and other specific causes (9 cases). The Kaplan-Meier plot of patient survival curves, grouped by the %LTM quartile, is shown in Fig 1. The overall 5-year patient survival rates were 50.2%, 55.3%, 61.0%, and 72.6% for patients with %LTM quartiles I-IV, respectively (log-rank test; *P* = 0.02) (Fig 1A). Univariate Cox regression analysis also showed that baseline %LTM was associated with patient survival (unadjusted hazard ratio, 0.980; *P* = 0.001) (Table S1). The impact on patient survival remained similar when male and female patients were analyzed separately (details not shown). The multivariable Cox regression model with backward stepwise analysis confirmed that only the baseline %LTM and Charlson comorbidity index were associated with patient survival (Table 4). In this model, every 1% increase in %LTM was associated with a 1.8% (95% confidence interval, 0.1%-3.4%) reduction in mortality.

**Table 4:** Multivariable Cox Regression Survival Models

Factors	AHR	95% CI	*P* Value
Patient survival			
%LTM	0.982	0.966-0.999	0.04
Charlson comorbidity score	1.221	1.127-1.324	<0.001
Peritonitis-free survival			
%LTM	0.983	0.968-0.998	0.03
Age	1.031	1.012-1.051	0.002
Fasting plasma glucose	1.097	1.010-1.192	0.03
Total cholesterol	0.860	0.742-0.996	0.04

*Note*: AHR represents per 1% change in %LTM, per 1 year in age, per 1 mmol/L in fasting plasma glucose or total cholesterol.

Abbreviations: AHR, adjusted hazard ratio; CI, confidence interval; %LTM, lean tissue mass percentage.

### Technique Survival

**Figure 2:**
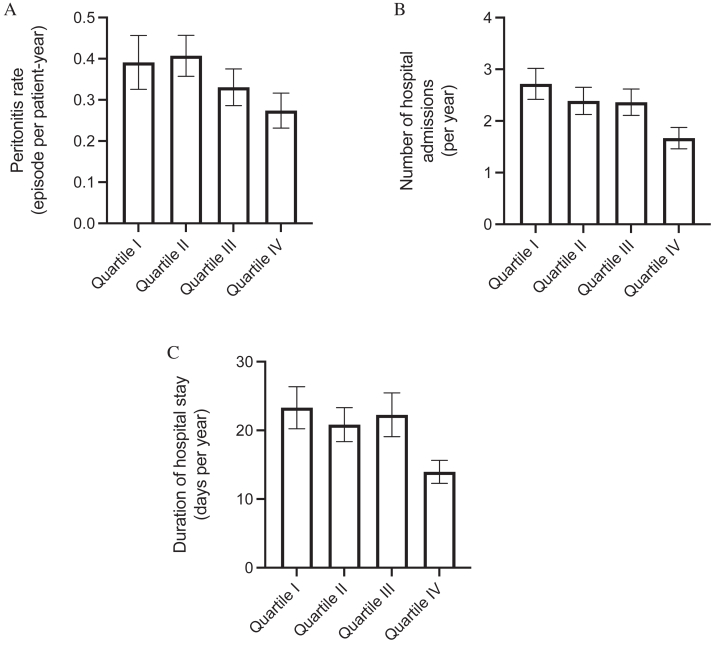
The relation between percentage of lean tissue mass (%LTM) quartiles and (A) peritonitis rate, (B) number of hospital admissions, and (C) duration of hospitalization. Quartile I has the lowest values. Error bars denote the standard error of the mean. Data were compared using the Jonckheere-Terpstra test.

During the study period, 44 patients switched to hemodialysis, 27 received kidney transplants, and 9 were transferred to other renal centers. The Kaplan-Meier plot of technique survival curves, grouped based on %LTM quartiles, is shown in Fig 2. The overall 5-year technique survival rates were 41.3%, 46.2%, 51.0%, and 54.1% for patients with %LTM quartiles I-IV, respectively (log-rank test; *P* = 0.29) (Fig 1B). Univariate Cox regression analysis also showed that baseline %LTM was not associated with technique survival (unadjusted hazard ratio, 0.991; *P* = 0.09) (Table S1).

### Peritonitis and Hospitalization

During the study period, there were 607 peritonitis episodes in 236 patients; 113 patients were peritonitis-free. The Kaplan-Meier plot of peritonitis-free survival curves, grouped based on %LTM quartiles, is shown in Fig 1C. The overall 5-year peritonitis-free survival rates were 13.8%, 13.3%, 26.2%, and 34.5% for patients with %LTM quartiles I-IV, respectively (log-rank test; *P* = 0.02) (Fig 1C). Univariate Cox regression analysis also showed that baseline %LTM was associated with peritonitis-free survival (unadjusted hazard ratio, 0.984; *P* = 0.005). The multivariable Cox regression model with backward stepwise analysis confirmed that only baseline %LTM, age, fasting plasma glucose, and total cholesterol level were associated with peritonitis-free survival (Table 4). The total peritonitis rates for quartiles I-IV of baseline %LTM were 0.39, 0.41, 0.33, and 0.27 episodes per patient-year, respectively (*P* = 0.16) (Fig 2A).

During the study period, there were 3,734 hospital admissions for a total of 31,627 days. The number of hospital admissions was 2.72, 2.39, 2.36, and 1.67 per year of follow-up for quartiles I-IV of baseline %LTM (*P* = 0.002) (Fig 2B), and the duration of hospital stay was 23.31, 20.84, 22.27, and 13.96 d/y for quartiles I-IV of baseline %LTM (*P* = 0.02) (Fig 2C). The relations among baseline LTM, %LTM, and adipose tissue mass, and peritonitis rate, hospitalization rate, and duration of hospitalization are summarized in Table S2. In short, baseline %LTM (but not baseline LTM) had modest but statistically significant negative correlations with the hospitalization rate (r = −0.173; *P* < 0.001) and the duration of hospitalization (r = −0.158; *P* < 0.001).

## Discussion

In the present study, we investigated the roles of baseline %LTM as the prognostic indicator of Chinese PD patients. Consistent with the original hypothesis, we found that the OH volume had a modest but significant positive correlation with LTM but not %LTM. In fact, %LTM had a significant inverse correlation with the extracellular-to-intracellular fluid volume ratio. We believe this is the result of mathematical coupling because skeletal muscle means is a major component of intracellular water, which is the denominator of the extracellular-to-intracellular fluid volume ratio. Taken together, our observation supports the notion that %LTM is the assessment of skeletal muscle mass without the interference of OH.

In this study, we found that %LTM was associated with patient survival, but not technique survival, in incident PD patients, whereas the absolute LTM value did not have a prognostic value. Consistent with our hypothesis, the absolute LTM did not predict patient survival, possibly because it was partly correlated with the OH volume, which was associated with higher mortality.^8^ Our finding is in line with the previous study of Lin et al,^16^ which showed that a high lean tissue index, but not body mass index or fat tissue index, predicted a lower risk of the composite outcome of death or cardiovascular events in patients with nondialysis-dependent chronic kidney disease. On the other hand, Marcelli et al^17^ reported that the survival of hemodialysis patients was best in those with a lean tissue index between the 10th and 90th percentiles of a healthy population. The discrepancy in the result may be due to the difference in dialysis modality or the average body built of the study populations. The relation between the comorbidity index and mortality is consistent with previous studies.^18^ On the other hand, several other factors have a role in predicting that the patient survival rate or other clinical outcomes in PD patients (eg, serum albumin level) did not have a significant effect in our multivariable regression model, probably because of the relatively small sample size of our cohort and collinearity of many parameters.

We observed a relation between %LTM and peritonitis-free survival but not the overall peritonitis rate. Our result appeared to be similar to that of the previous report of Do et al,^19^ which showed that low skeletal muscle mass was associated with increased risks of peritonitis. Nonetheless, there are important differences between the studies. Do et al^19^ used absolute skeletal muscle mass, a specific cutoff level for low muscle mass, for binary analysis, and patients were grouped based on with and without peritonitis, whereas the peritonitis rate was not reported. On the other hand, we used %LTM as a continuous variable for correlational analysis. Furthermore, absolute LTM was not associated with peritonitis-free survival or overall peritonitis rate in our analysis (see Tables S1 and S2). Further studies are much needed to investigate the relation between muscle built and the risk of PD-related peritonitis. We also found that a high fasting-glucose level and a low total cholesterol level were associated with peritonitis events, which is consistent with the results of previous studies.^20^^,^^21^

We observed that baseline %LTM was associated with the subsequent hospitalization rate and the duration of hospitalization, whereas the absolute LTM did not predict the hospitalization rate. Our finding is consistent with a previous study in hemodialysis, which reported that sarcopenia was associated with a higher hospitalization rate.^22^ In patients without kidney failure, lower muscle mass was reported to be associated with the number of hospitalizations.^23^ Although we are unclear why the absolute LTM did not show such a relation, the relationship between %LTM and hospitalization emphasizes the importance of monitoring body composition in dialysis practice. Further studies are warranted to explore the underlying mechanisms linking %LTM with hospitalization and evaluate potential interventions for preserving LTM in dialysis patients.

This study has several limitations. First, although we studied the roles of %LTM in predicting a panel of outcome measures, including patient survival, technique survival, peritonitis-free survival, and hospitalization rates, there are still important aspects that were not taken into consideration. Specifically, we did not analyze the contribution of cardiovascular incidents and infection to mortality and hospitalization. Unfortunately, because of the limitations in the original study design, we do not have other surrogate markers (eg, serum C-reactive protein and brain natriuretic peptide levels), which may provide additional insights into the contribution of systemic inflammation and cardiac stress to clinical outcomes. Studies of substantially larger sample sizes would be needed to answer this question. Second, despite the observation of a significant prognostic implication of %LTM, we did not determine a cutoff value to define the high-risk group that had an increased risk of adverse clinical outcomes. In fact, the correlation and regression approach of our analysis assumed that the relation between %LTM and outcome parameters is linear, which may or may not be the case. Further research is needed to delineate the type of relation between %LTM and the outcome of dialysis patients and to define the cutoff value of %LTM (if there is one) for the diagnosis of sarcopenia.

In conclusion, our study showed that baseline %LTM as measured using BIS is associated with patient survival, peritonitis-free survival, the number of hospital admissions, and the duration of hospital stay in incident PD patients. Further studies are needed to determine the underlying mechanisms and to define the cutoff value of %LTM for the diagnosis of sarcopenia.
