# Supplementary material for: Prognostic Significance of Baseline Lean Tissue Mass Percentage in Incident Peritoneal Dialysis Patients
Source: Kidney Med. 2026 Jan 10;8(3):101260. doi: 10.1016/j.xkme.2026.101260 (PMC12936936; doi:10.1016/j.xkme.2026.101260)
Supplement: Supplementary File (PDF) — Tables S1, S2. [file mmc1.pdf]

Table S1. Univariate Cox regression analysis for patient, technique, and peritonitis-free survival

	Patient survival		Peritonitis-free survival		Technique survival	
	Exp (B)	P values	Exp (B)	P values	Exp (B)	P values
Sex	1.168	0.38	1.164	0.31	1.253	0.14
Age	1.049	< 0.001	1.030	< 0.001	1.015	0.03
Height	0.997	0.73	1.000	0.99	1.016	0.09
Weight	1.010	0.09	1.019	0.004	1.020	< 0.001
Blood pressure						
Systolic	1.004	0.33	0.999	0.77	1.004	0.22
Diastolic	0.982	0.007	0.988	0.04	0.994	0.26
Charlson’s comorbidity score	1.249	< 0.001	1.130	< 0.001	1.177	< 0.001
Bioimpedance spectroscopy						
LTM	0.990	0.22	0.998	0.75	1.010	0.18
%LTM	0.980	0.001	0.984	0.005	0.991	0.09
ATM	1.013	0.07	1.019	0.01	1.012	0.09
Overhydration volume	1.122	< 0.001	1.092	< 0.001	1.113	< 0.001
E/I ratio	16.788	< 0.001	6.411	< 0.0001	5.893	< 0.001
Haemoglobin	0.885	0.05	1.018	0.72	0.963	0.49
Serum albumin	0.950	0.005	0.972	0.06	0.972	0.08
Fasting plasma glucose	1.125	0.009	1.072	0.07	1.132	0.001
Lipid profile						

Total cholesterol	0.884	0.10	0.886	0.07	0.919	0.18
LDL	0.894	0.21	0.898	0.17	0.929	0.34
HDL	0.661	0.11	0.723	0.11	0.760	0.21
Triglyceride	1.046	0.62	0.992	0.92	1.013	0.88
Total weekly Kt/V	0.772	0.19	0.858	0.35	0.678	0.04
Residual GFR	0.992	0.85	1.048	0.16	0.998	0.97
Iron profile						
Plasma iron	0.954	0.02	0.982	0.29	0.964	0.04
Plasma TIBC	0.975	0.07	0.999	0.94	0.986	0.21
Iron saturation	0.404	0.17	1.163	0.77	0.432	0.14
Serum ferritin	1.000	0.23	1.000	0.10	1.000	0.96

OH, overhydration; E/I, extra to intracellular volume ratio; LTM, lean tissue mass; %LTM, lean tissue mass percentage; LTI, lean tissue index; ATM, adipose tissue mass; GFR, glomerular filtration rate; LDL, low-density lipoprotein; HDL, high-density lipoprotein; TChol, total cholesterol; TIBC, total iron binding capacity.

Table S2. Correlation between baseline bioimpedance spectroscopy measurements and other clinical and biochemical parameters with the rate of hospitalisation, duration of hospitalisation, and peritonitis rate

	no. of peritonitis episode*		no. of hospital admission*		duration of hospital stay*	
	r	P values	r	P values	r	P values
Bioimpedance spectroscopy measurements						
LTM	-0.095	0.08	-0.043	0.42	-0.039	0.47
%LTM	-0.078	0.15	-0.173	0.001	-0.158	0.003
ATM	0.028	0.60	0.152	0.005	0.135	0.01
Overhydration	0.033	0.54	0.150	0.005	0.159	0.003
E/I ratio	0.081	0.13	0.229	< 0.001	0.231	< 0.001
Sex	-0.014	0.80	0.103	0.06	0.093	0.08
Age	0.111	0.04	0.299	< 0.001	0.289	< 0.001
Height	0.001	0.98	0.013	0.81	0.017	0.76
Weight	-0.051	0.34	0.119	0.03	0.115	0.03
Blood pressure						
Systolic	0.005	0.92	0.048	0.37	0.080	0.14
Diastolic	-0.034	0.53	-0.137	0.01	-0.117	0.03
Charlson's comorbidity score	0.113	0.04	0.345	< 0.001	0.319	< 0.001

Haemoglobin	-0.016	0.77	-0.151	0.005	-0.077	0.16
Serum albumin	-0.131	0.02	-0.212	< 0.001	-0.224	< 0.001
Fasting plasma glucose	0.101	0.114	0.170	0.008	0.147	0.02
Lipid profile						
Total Cholesterol	-0.037	0.56	-0.070	0.27	-0.090	0.16
LDL	-0.001	0.99	-0.009	0.89	-0.026	0.69
HDL	-0.035	0.59	-0.130	0.05	-0.159	0.01
Triglyceride	-0.026	0.69	0.023	0.72	0.038	0.55
Total Weekly Kt/V	-0.006	0.94	-0.020	0.78	-0.020	0.78
Residual GFR	-0.057	0.43	0.049	0.50	0.066	0.36
Iron profile						
Plasma iron	0.106	0.10	-0.037	0.56	-0.036	0.57
Plasma TIBC	-0.021	0.74	-0.160	0.01	-0.163	0.01
Iron saturation	0.110	0.09	0.033	0.61	0.042	0.52
Ferritin	0.042	0.53	0.110	0.10	0.107	0.11

*adjusted for the duration of follow-up; data were compared by Spearman's rank sum test.

LTM, lean tissue mass; %LTM, lean tissue mass percentage; ATM adipose tissue mass; E/I, extra to intracellular volume ratio; LDL, low-density lipoprotein; HDL, high-density lipoprotein; GFR, glomerular filtration rate; TIBC, total iron binding capacity
